# Remote Management of Poststroke Patients With a Smartphone-Based Management System Integrated in Clinical Care: Prospective, Nonrandomized, Interventional Study

**DOI:** 10.2196/15377

**Published:** 2020-02-27

**Authors:** Do Yeon Kim, Hee Kwon, Ki-Woong Nam, Yongseok Lee, Hyung-Min Kwon, Young Seob Chung

**Affiliations:** 1 Department of Neurology Seoul National University College of Medicine Seoul National University Hospital Seoul Republic of Korea; 2 Medical Corps Republic of Korea Navy Jeju Island Republic of Korea; 3 LifeSemantics, Corp Seoul Republic of Korea; 4 Department of Neurology Seoul National University College of Medicine Seoul Metropolitan Government-Seoul National University Boramae Medical Center Seoul Republic of Korea; 5 Department of Neurosurgery Seoul National University College of Medicine Seoul Metropolitan Government-Seoul National University Boramae Medical Center Seoul Republic of Korea

**Keywords:** mHealth, mobile apps, stroke care, health care, patient education, self-monitoring of blood pressure

## Abstract

**Background:**

Advances in mobile health (mHealth) have enabled systematic and continuous management of patients with chronic diseases.

**Objective:**

We developed a smartphone-based mHealth system and aimed to evaluate its effects on health behavior management and risk factor control in stroke patients.

**Methods:**

With a multifaceted stroke aftercare management system that included exercise, medication, and educational materials, we performed a 12-week single-arm intervention among eligible poststroke patients in the stroke clinic from September to December 2016. The intervention consisted of (1) regular blood pressure (BP), blood glucose, and physical activity measurements; (2) stroke education; (3) an exercise program; (4) a medication program; and (5) feedback on reviewing of records by clinicians. Clinical assessments consisted of the stroke awareness score, Beck Depression Inventory-II (BDI), EuroQol-5 Dimensions (EQ-5D), and BP at visit 1 (baseline), visit 2 (4 weeks), and visit 3 (12 weeks). Temporal differences in the parameters over 12 weeks were investigated with repeated-measures analysis of variance. Changes in medication adherence at visit 1-2 (from visit 1 to visit 2) and visit 2-3 (from visit 2 to visit 3) were compared. System satisfaction was evaluated with a self-questionnaire using a 5-point Likert scale at visit 3.

**Results:**

The study was approved by the Institutional Review Board in September 2016, and participants were enrolled from September to December 2016. Among the 110 patients enrolled for the study, 99 were included in our analyses. The mean stroke awareness score (baseline: 59.6 [SD 18.1]; 4 weeks: 67.6 [SD 16.0], *P*<.001; 12 weeks: 74.7 [SD 14.0], *P*<.001) and BDI score (baseline: 12.7 [SD 10.1]; 4 weeks: 11.2 [SD 10.2], *P*=.01; 12 weeks: 10.7 [SD 10.2], *P*<.001) showed gradual improvement; however, no significant differences were found in the mean EQ-5D score (baseline: 0.66 [SD 0.33]; 4 weeks: 0.69 [SD 0.34], *P*=.01; 12 weeks: 0.69 [SD 0.34], *P*<.001). Twenty-six patients who had uncontrolled BP at baseline had −13.92 mmHg (*P*=.001) and −6.19 mmHg (*P*<.001) reductions on average in systolic and diastolic BP, respectively, without any antihypertensive medication change. Medication compliance was better at visit 2-3 (60.9% [SD 37.2%]) than at visit 1-2 (47.8% [SD 38.7%], *P*<.001).

**Conclusions:**

Awareness of stroke, depression, and BP was enhanced when using the smartphone-based mHealth system. Emerging mHealth techniques have potential as new nonpharmacological secondary prevention methods because of their ubiquitous access, near real-time responsiveness, and comparatively lower cost.

## Introduction

Recurrent stroke accounts for approximately 30% of all stroke events and causes greater mortality, disability, and economic burden when compared with first-ever stroke [1-4]. The cumulative risk of stroke recurrence in stroke survivors is on average 11.1% at 1 year and 26.4% at 5 years [5]. Recurrent stroke is largely associated with vascular risk factor burden, and therefore, current stroke prevention has focused on developing multidisciplinary approaches to control hypertension, diabetes mellitus, dyslipidemia, obesity, and physical inactivity [6,7]. As poststroke management is becoming a lifelong process, easily accessible, reciprocal, and low-cost supportive tools are required for stroke patients to control modifiable risk factors and maintain secondary prevention on a regular and extended basis.

Advances in mobile health (mHealth) have enabled remote monitoring and management that were otherwise confined to health centers. The advantages of mHealth technology include ubiquitous access, near real-time responsiveness, and comparatively lower cost when compared with conventional outpatient management [8,9]. These positive factors match the requisites of an ideal stroke prevention tool. In the period of telephone and Web-based poststroke care [10-12], an mHealth platform for stroke patients has been studied, and the potential advantages of an mHealth app have been suggested for some outcomes including blood pressure (BP) and medication adherence [13]. This indicates that a multifunctional mHealth platform targeting broader and more diverse outcomes, including depression and quality of life, which were found to be affected in a telephone or Web-based management system, is needed [14].

Complete understanding and proper awareness of stroke are essential for stroke survivors, as stroke awareness is related to in-time treatment of stroke through a decrease in prehospital delay [15]. Stroke awareness among stroke patients includes awareness about the definition, risk factors, and treatment of stroke, and the action plan against stroke symptoms [16]. Stroke patients who understand the risk factors and treatment of stroke well may adjust their lifestyle cautiously, maintain their treatment confidentially, and, more importantly, initiate acute stroke treatment as soon as possible in case of recurrence. An mHealth app that offers extensive information on stroke and an interactive education program to patients would improve their awareness of the risk factors and symptoms. Therefore, it is essential to determine whether the stroke awareness of patients improves after using an mHealth app.

mHealth apps that aid in BP control and medication adherence have been reported to improve outcomes in patients with chronic diseases [17-20]. As BP control is a key aspect of secondary stroke prevention, mHealth apps could be applied to efficiently maintain BP with a regular BP check and with exercise and medication monitoring on a daily basis. For stroke survivors, adherence to multiple drug regimens, including antiplatelet, antihypertensive, antidiabetic, and lipid-lowering agents, is essential for secondary prevention, and mHealth apps could be applied to monitor and encourage the medication intake of patients.

As mentioned above, we hypothesized that a multifaceted mHealth platform would improve stroke awareness, mood, and quality of life, as well as support risk factor control in poststroke patients. The aim of this study was to develop a multifunctional mHealth platform that could manage posthospital stroke patients integrated in clinical care and to investigate changes in stroke awareness, mood, and quality of life; adherence to app use; and satisfaction with the system after intervention among stroke patients. The study also endeavored to investigate the effects of mHealth app use on BP control and other physical measurements in stroke patients, as it has been suggested to be beneficial in patients with other chronic diseases.

## Methods

### Mobile Health Care System: Smart Aftercare

**Figure 1:**
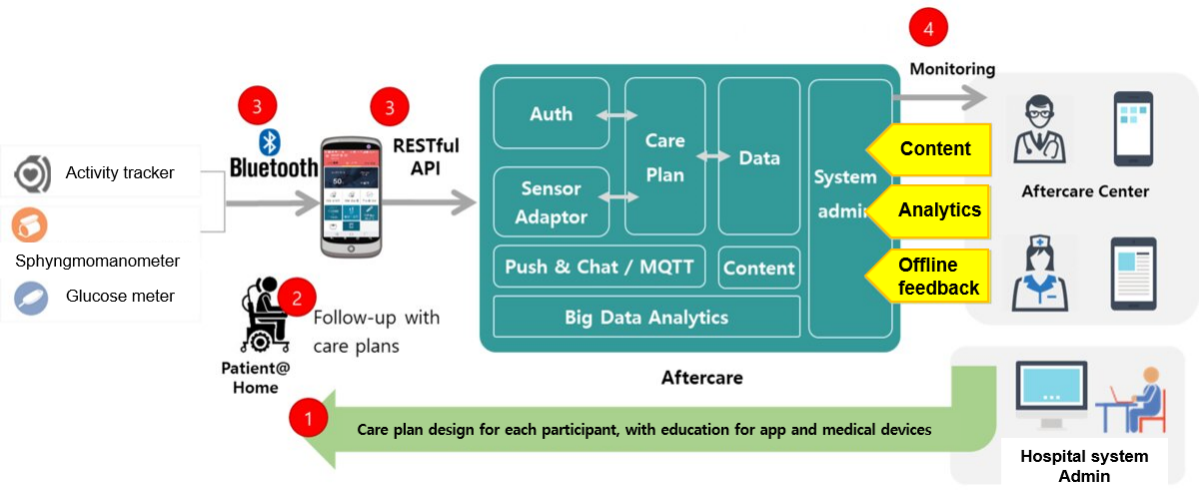
Schematic view of Smart Aftercare. MQTT: message queue for telemetry transport.

Smart Aftercare takes a mobile-based holistic approach, and it includes wearable devices, a personalized poststroke management app, and a server-side website for patient monitoring by clinicians (Figure 1). Participants were provided with a Bluetooth sphygmomanometer (A&D UA-651BLE, A&D Engineering, Inc, San Jose, California) and a wrist-worn smart band (activity tracker; Croise S, Partron Co, Ltd, Gyeonggi-do, Republic of Korea), and patients with diabetes used a glucose meter (CareSens N, i-sens Inc, Seoul, Republic of Korea). BP, blood glucose levels, and physical activity records were transmitted to a central site for clinicians to review and act upon.

### Multifaceted Mobile Management for Stroke Patients

**Figure 2:**
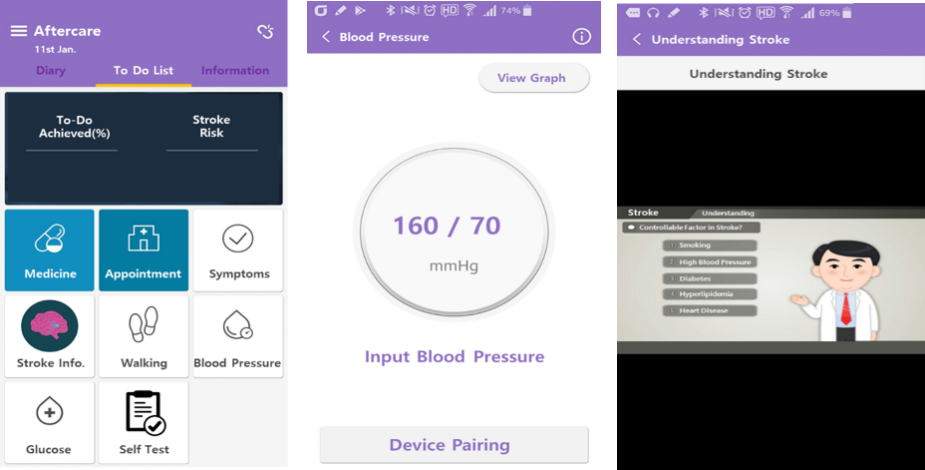
Image showing the entry screen (left), blood pressure recording (middle), and educational content (right).

The mobile app supports stroke patients with various health management functions as follows: management and monitoring of medication, clinic visit schedule, stroke education program, self-testing of stroke symptoms, exercise program, and BP, blood glucose, and physical activity measurements (Figure 2).

Management and monitoring of medication are essential functions of an mHealth app for chronic diseases. Adherence to antihypertensive medication was found to be dose-dependently associated with a low stroke risk in a previous study [21], and persistence with antiplatelet therapy was found to be associated with a 72.5% lower likelihood of recurrent ischemic stroke [22]. Therefore, an mHealth app for the prevention of stroke needs medication management for monitoring and encouraging intake of drugs, including antiplatelet and antihypertensive agents. The medication management of the app includes medication alarms, prescription information, and registration of intake and medication adverse effects, if they occur.

Awareness of stroke and self-testing of stroke symptoms are related to early arrival at the hospital, which is a critical factor for increasing the efficacy of thrombolysis therapy [23]. With these functions, the mHealth app could contribute to the enhancement of stroke outcomes. The stroke education program module offers stroke patients a self-assessment of stroke symptoms, a weekly updated newsfeed about stroke, answers to frequently asked questions, and exercise recommendations for stroke prevention.

Several previous smartphone usage studies about the physical activity influence reported that physical activity increases (by 800-1104 steps/day) [24]. The exercise program module connected with the smart band includes step count, moving distance, consumed calories, exercise time, and heart rate during exercise. These values are recorded and subsequently reviewed. The efficiency of the workout is determined by the intensity of walking, which is assessed by the heart rate increment and walking speed. The app also provides information on muscular exercises and stretching instructions, which are updated monthly. Daily exercise tasks are assigned to the users and exercise goal achievement rates are recorded.

There have been several randomized controlled trials on the efficacy of mHealth technology to promote BP control for cardiovascular disease prevention [25,26]. Self-measurement of BP with an mHealth app has been shown to improve BP control in patients with uncontrolled hypertension [27]. Studies using mHealth technology for glucose control reported a more than 1% greater hemoglobin A1c decline in an intervention group that received summarization of glycemic control, diabetes medication management, and information on lifestyle behaviors with current treatment, when compared with the finding in a control group that received medical treatment only [28,29]. The integrated exercise program, BP management, and glucose management functions may contribute to efficient health behavior changes, BP control, and glucose control. According to the abovementioned literature, data on BP and blood glucose are saved in the system and reviewed by participants and clinicians. App reminders notify about medication intake (activated at the prescribed time), BP assessment (activated twice a day [7 am and 9 pm]), and blood glucose assessment (activated as individually set at the first visit by the clinician) (Multimedia Appendix 1).

### Patient Management Website

**Figure 3:**
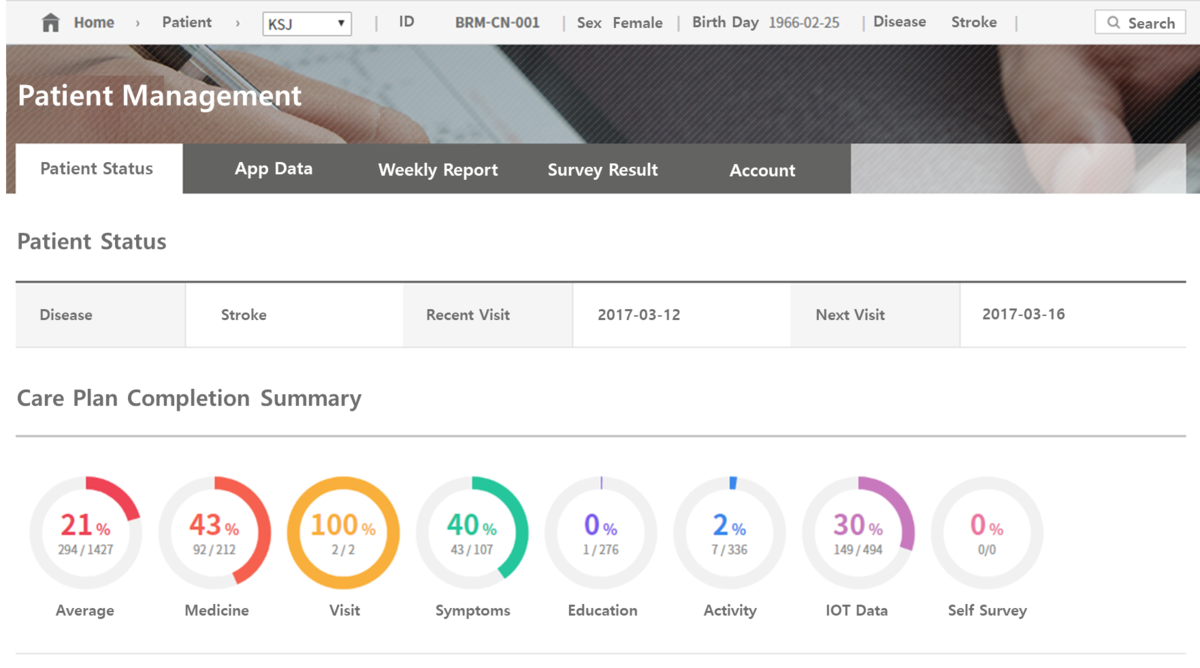
Summary of care plan completion on the patient management system.

The website for clinicians stores and displays the patient’s health record generated and sent from the app. Strict access control is in place for the secure database so that only authorized clinicians can view patient data. The site provides a summary of the health progress and status of each patient registered. Figure 3 shows records of medication, reported symptoms, viewed education content, and clinic visits.

### Study Design

After the development of an mHealth care system, a 12-week single-arm intervention was performed with eligible poststroke patients from Seoul Metropolitan Government-Seoul National University Boramae Medical Center. The inclusion criteria were as follows: (1) diagnosis of stroke (including ischemic and hemorrhagic stroke) supported by clinical symptoms and brain imaging; (2) age >19 years; (3) agreement to sign a written informed consent form; and (4) adequate ability to use a smartphone (either the patient or the guardian). Candidates who were fully dependent on caregivers owing to stroke sequelae were excluded from the study (modified Rankin Scale score of 4 or 5) [30].

The intervention comprised the following: (1) measurements of regular BP (twice a day [7 am and 9 pm]), blood glucose (as decided in the clinic), and physical activity (with the smart band); (2) stroke education program module; (3) exercise program module (exercise and stretching education); (4) medication management; and (5) feedback from the patient to the clinician with review of the health records registered in the system. This study was approved by the Institutional Review Board (IRB) at Seoul Metropolitan Government-Seoul National University Boramae Medical Center (IRB #16-2016-98).

The participants had three visits to the clinic (visit 1: baseline, visit 2: 4 weeks from baseline, and visit 3: 12 weeks from baseline) within a span of 12 weeks. At baseline (visit 1), eligibility for the study was determined according to previous medical history, medication history, neurological examination findings, and the modified Rankin scale score, and signed consent was obtained from each participant. Stroke awareness, depression scale scores, and health-related quality of life (HRQoL) were determined at each visit. Physical measurements, including height, weight, body mass index (BMI), waist circumference, and systolic and diastolic BP (SBP and DBP), were checked at each visit. System utilization was checked at the end of the intervention using saved app data. System satisfaction was assessed at visit 3 using a structured self-questionnaire.

### Outcome Measures

#### System Utilization and System Satisfaction

Individual utilization of the programs was defined by the average amount of program access during the intervention, which was assessed using the logged data of the mobile app. System satisfaction was evaluated using a 5-point Likert scale, which was calculated from the participants’ responses on their level of agreement or disagreement after 12 weeks (1, strongly disagree; 2, disagree; 3, neither agree nor disagree; 4, agree; or 5, strongly agree) for overall system satisfaction and on satisfaction subscales (satisfaction of system information, wish to continue the program after the study, wish to introduce the app to others, interest in their health, and reliance on clinicians).

#### Stroke Awareness, Depression, and Health-Related Quality of Life

Multimedia Appendix 2 summarizes the clinical outcomes according to each assessment criterion. Patients’ awareness of stroke was measured according to the stroke awareness score, which evaluates knowledge of stroke and ability to cope when stroke symptoms occur [16]. The stroke awareness score consists of the following four parts: definition of stroke, risk factors of stroke, treatment of stroke, and action plan against stroke (Multimedia Appendix 3). The score of each part was calculated as a percentage. Beck Depression Inventory-II (BDI) for depression and EuroQol-5 Dimensions (EQ-5D) for HRQoL were evaluated using questionnaires at each visit [31].

#### Physical Measurements

SBP, DBP, BMI, and waist circumference were measured at each visit. The participants were divided into the following two groups: one with SBP >140 mmHg or DBP >90 mmHg and the other with BP in the normal range at visit 1, and the differential effects of the system in patients with uncontrolled high BP and those with BP in the normal range were investigated.

### Statistical Analysis

Temporal differences in the stroke awareness, BDI, and EQ-5D scores over 12 weeks were investigated using repeated-measures analysis of variance. The effects of the system were compared between patients with initial BDI scores indicative of depression (BDI ≥14 points) and those without depression. Temporal changes in physical measurements, including SBP, DBP, BMI, weight, and waist circumference, were analyzed using repeated-measures analysis of variance. Changes in medication adherence at visit 1-2 (from visit 1 to visit 2) and visit 2-3 (from the day after visit 2 to visit 3) were analyzed using the paired *t* test. All analyses were performed using R software, version 3.1.1 (R Foundation for Statistical Computing, Vienna, Austria).

## Results

### Participant Characteristics

**Figure 4:**
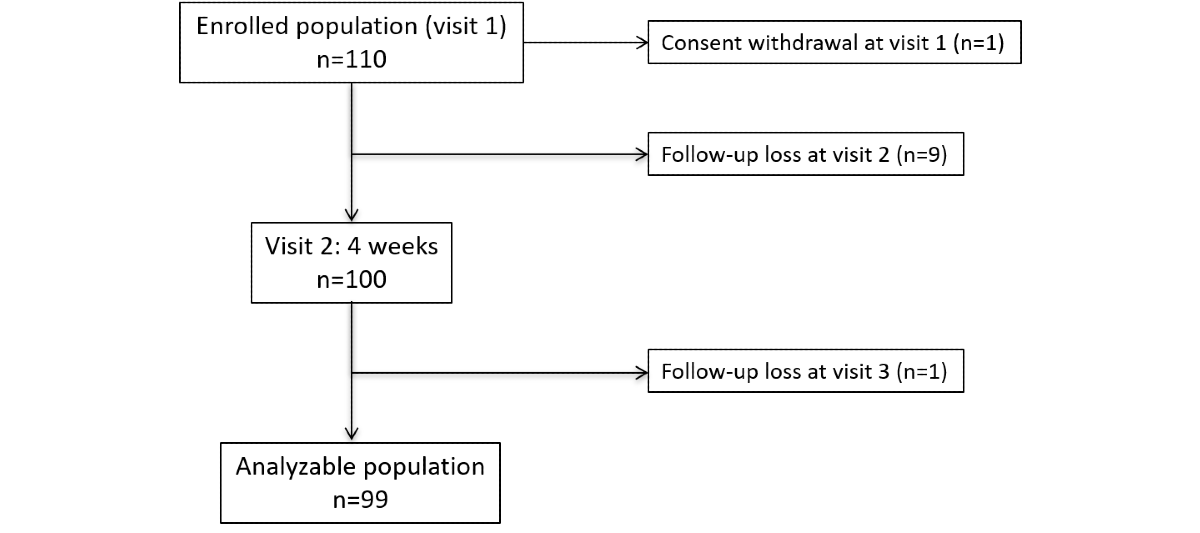
Study flow chart.

This study was approved by the IRB in September 2016, and it enrolled participants from September 2016 to December 2016. A total of 110 patients were enrolled for this study. Of the enrolled patients, one pulled out from the study at visit 1. Additionally, nine patients did not return to the clinic at visit 2 and one patient did not come for visit 3. Thus, 99 patients were included in our analysis (Figure 4). The mean time since stroke among the patients was 40.5 (SD 48.7) months. The baseline characteristics of the participants are described in Multimedia Appendix 4. Among the 99 patients, 61 had ischemic stroke and 38 had hemorrhagic stroke, of which 30 had intracerebral hemorrhage and 8 had subarachnoid hemorrhage. As for the underlying vascular risk factors, 71 patients had hypertension, 20 had diabetes, and 32 had hyperlipidemia.

### System Utilization and System Satisfaction

The mean access numbers of the mobile app during the follow-up period were 100.9 for medication intake, 24.0 for the exercise program, 90.6 for BP measurement, and 29.1 for stroke education content. In the 5-point system satisfaction survey, the mean overall satisfaction score and satisfaction score for system information were 3.74/5 and 3.81/5, respectively, which indicated a positive result for satisfaction. Participants wished to continue the program after the study (3.98/5) and were willing to introduce the app to others (4.06/5). Increments in the level of interest in their health (4.02/5) and reliance on clinicians (4.08/5) were observed.

### Awareness of Stroke, Depression, and Health-Related Quality of Life

**Figure 5:**
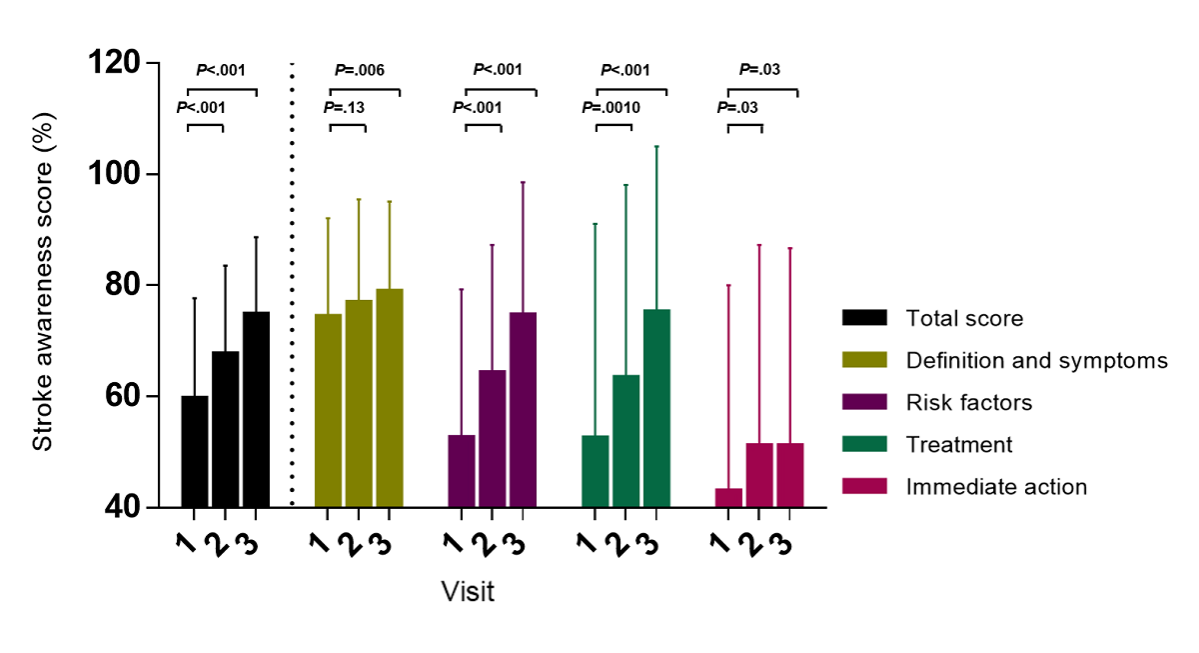
Trends in the stroke awareness score and its four components using the smartphone-based management system.

The stroke awareness score of the participants showed a gradual improvement in the aptitude of using the program by 7.98% in 4 weeks (*P*<.001) and 15.12% in 12 weeks (*P*<.001), as shown in Multimedia Appendix 5. In detail, knowledge about the immediate actions against stroke, definition and symptoms of stroke, and treatment and risk factors of stroke were enhanced after the intervention (Figure 5).

**Figure 6:**
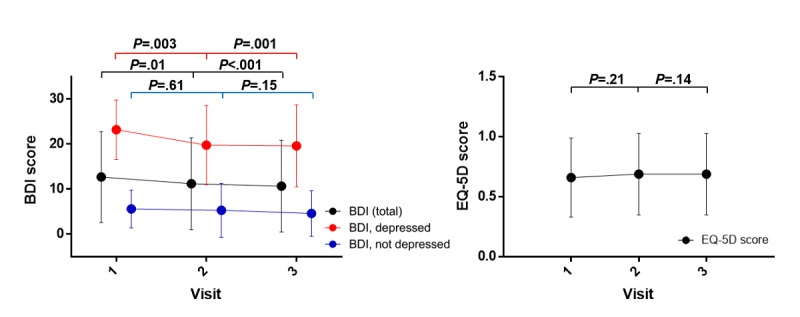
Trends of the Beck Depression Inventory-II (BDI) score in patients who were depressed and not depressed (left) and of the EuroQol-5 Dimensions (EQ-5D) score (right).

Furthermore, the BDI scores decreased at visit 2 (−1.57, *P*=.01) and visit 3 (−2.07, *P*<.001) when compared with the score at visit 1, as shown in Multimedia Appendix 6. A significant decrease in the BDI score (by −3.63, *P*<.001) was observed solely in depressed patients (Figure 6); however, improvement in the EQ-5D score was not significant (Figure 6).

### Physical Measurements

**Figure 7:**
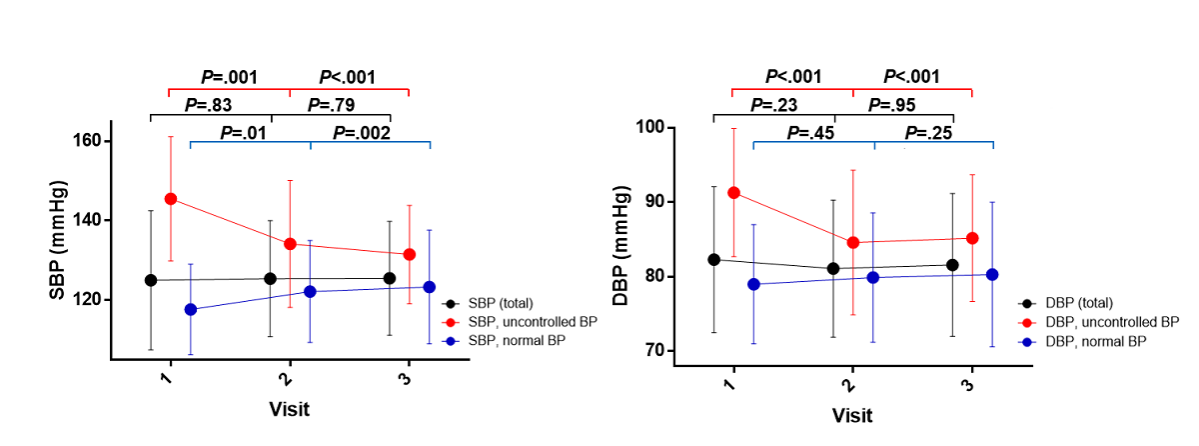
Different trends of systolic blood pressure (SBP) (left) and diastolic blood pressure (DBP) (right) reductions in patients with uncontrolled and normal blood pressure.

Among the 99 patients, 26 had SB*P*>140 mmHg or DB*P*>90 mmHg and the rest (n=73) had BP in the normal range. Significant drops in both SBP and DBP by averages of −13.92 (*P*<.001) and −6.19 mmHg (*P*<.001), respectively, were found in the high BP group without medication change over 12 weeks, as shown in Figure 7. In accordance with this change, compliance with medication improved at visit 2-3 (60.9% [SD 37.2%]) from visit 1-2 (47.8% [SD 38.7%]) (*P*<.001). BMI and waist circumference showed no significant decreases till the end of the intervention, as shown in Multimedia Appendix 7.

## Discussion

In this study, we demonstrated that the mobile health care system Smart Aftercare improved the level of stroke awareness and lowered the depression score among poststroke patients in sequential evaluations that were performed for 12 weeks, when compared with the findings at the start of the study. A multifaceted mHealth system that offered stroke education, medication and exercise management, BP management, blood glucose management, physical activity measurements, and clinician feedback according to patient data provided a high level of system satisfaction to patients and improved the levels of interest in their health and reliance on clinicians. Hypertensive patients at baseline benefitted from the system, with lowered SBP and DBP during the intervention without a change in antihypertensive medication. An improvement in medication compliance was found in accordance with this change.

The findings of this study suggest that mobile health care could enhance stroke awareness in stroke patients. Previous studies using mHealth technology aimed to facilitate BP control and compliance with medication for stroke [13,32,33]; however, this study targeted broader poststroke outcomes including stroke awareness, depression, HRQoL, and BP. Importance of stroke awareness has been reported to decrease prehospital delay in treatment after acute stroke [15]. Early arrival at the hospital is a critical factor for increasing the efficacy of intravenous thrombolysis administered within 3 hours in elderly patients aged >80 years and 4.5 hours in patients aged 18-80 years [23,34]. Proper stroke awareness, including knowledge of stroke symptoms, appropriate remedial actions, and understanding of time-sensitive treatment, is associated with better stroke outcomes. Smart Aftercare allows for increased stroke awareness with the help of daily stroke-related articles and videos, exercise methods for stroke prevention, and frequently asked questions and answers for stroke. With a sufficient level of stroke awareness, patients would be able to distinguish genuine stroke symptoms, which could help them to seek timely treatment.

The alleviated depression in stroke survivors using mHealth technology has practical importance in improving the well-being of poststroke patients. One-third of stroke survivors experience depression, and this has a negative effect on functional stroke outcomes, thus limiting participation in rehabilitation activities and impeding social function and adjustment [35,36]. Pharmacological therapy could be one of the treatment options for poststroke depression; however, nonpharmacological therapy has been receiving attention owing to possible adverse effects caused by antidepressants and potential drug interactions with anticoagulants [37]. In a recent clinical trial, a blended treatment using a mobile app in addition to conventional treatment methods demonstrated successful utilization of the mHealth app for managing depression, with a change in participants’ depressed behavior to healthy behavior [38]. Smart Aftercare might modify patients’ behavior toward proper health habits with the induction of regular exercise, compliance with medication intake, and monitoring of physical parameters. Awareness of stroke and continuous provision of precise information might reduce the unnecessary fear of stroke recurrence.

This study showed the system’s efficacy in controlling BP, a critical element in the prevention of secondary stroke. Self-measurement of BP has been proven to lower BP when compared with traditional center-based care in hypertensive patients [39]. This study reaffirms the applicability of mHealth technology to BP management, especially for patients with high BP [40]. The BP lowering effect without any medication change could be attributed to improved medication adherence with the use of mHealth technology, and this suggests that pharmacological treatment along with continuously monitored medication compliance is superior to medication alone. The American Heart Association also supports the role of mHealth technology in reducing BP, while pointing out the need for targeting broader stakeholders, including the elderly [25]. Considering the average age of patients in this study (57.9 years), this smartphone-based system showed the applicability of mHealth apps to old age groups. The medication reminder to urge patients to take antihypertensive and antithrombotic tablets, without a medication change during the study, is considered to play important roles in reducing high BP and the risk of thromboembolic events, respectively. Furthermore, clinicians can apply recorded BP data in medication adjustment and patient feedback; therefore, the power of mHealth technology can afford further effectiveness in medication management.

This study has several limitations. It was conducted in a single center and was a single-arm study. Owing to the nature of mHealth technology, patients with severe disability or without a smartphone were not included in this study. Furthermore, the effectiveness of the intervention was somewhat attenuated because the participants were treated under the current medical care in the clinic before the study, and therefore, the vital signs and anthropometric measurements, including BP and BMI, of most of the participants were already within the normal ranges at baseline.

### Conclusions

Use of Smart Aftercare, which enhances the level of awareness of stroke and depression, could spur a major shift in the planning of poststroke care after hospitalization. mHealth technology with multifaceted programs and responsive capacities might enable feasible, immediate, and efficient poststroke home care and might consequently contribute to cost-effective secondary stroke prevention.

## Appendix

Multimedia Appendix 1 Screenshots of the intervention.

Multimedia Appendix 2 Clinical assessment of outcomes in this study.

Multimedia Appendix 3 Questionnaire of the stroke awareness score in English.

Multimedia Appendix 4 Demographic characteristics and clinical information of the study participants.

Multimedia Appendix 5 Changes in the total stroke awareness score and the scores of each part using the smartphone-based management system.

Multimedia Appendix 6 Changes in the Beck Depression Inventory-II (BDI) scores among patients who were depressed and not depressed and in the EuroQol-5 Dimensions (EQ-5D) scores.

Multimedia Appendix 7 Changes in blood pressure, body mass index, and waist circumference.

## Abbreviations

BDI: Beck Depression Inventory-II
BMI: body mass index
BP: blood pressure
DBP: diastolic blood pressure
EQ-5D: EuroQol-5 Dimensions
HRQoL: health-related quality of life
IRB: Institutional Review Board
SBP: systolic blood pressure
