# Supplementary material for: Remote Management of Poststroke Patients With a Smartphone-Based Management System Integrated in Clinical Care: Prospective, Nonrandomized, Interventional Study
Source: J Med Internet Res. 2020 Feb 27;22(2):e15377. doi: 10.2196/15377 (PMC7068458; doi:10.2196/15377)

Multimedia Appendix 1. Screenshots of the intervention

Self-testing of stroke symptoms

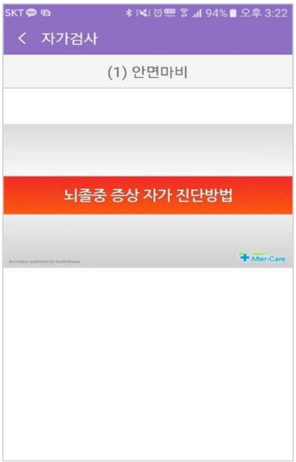

Registration of medication intake

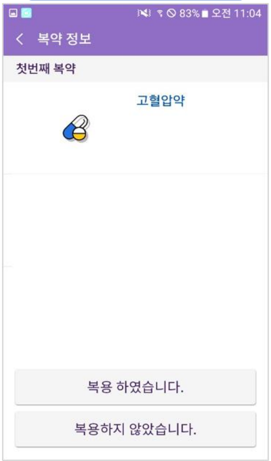

Stroke education: post-stroke home-rehabilitation

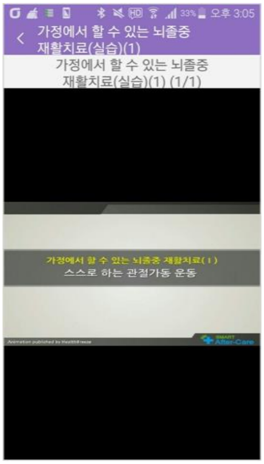

Stroke education: daily newsfeed

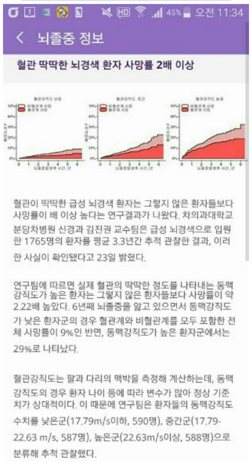

Exercise program : evaluation after exercise

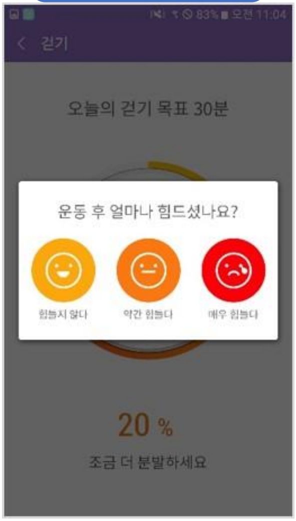

Exercise program : Instructions for stretching

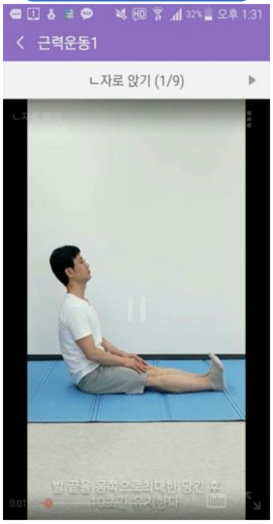

Exercise program : Alarm for too slow walking speed

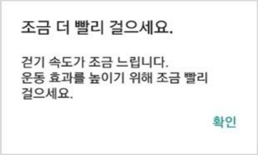

Weekly report for participants

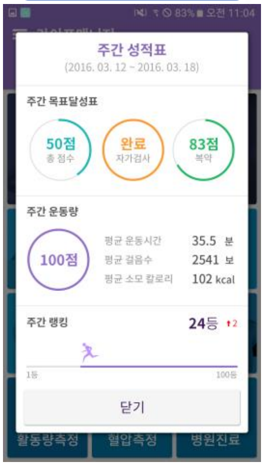

Exercise program : Summary of walking steps (green), consumed calories (red), walking distance (blue)

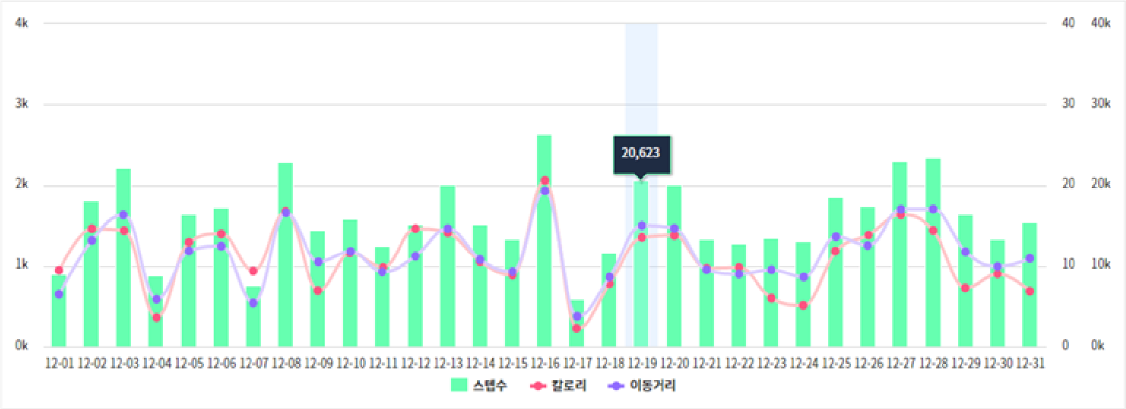

Smart Band and exercise program:
walking mode screenshot

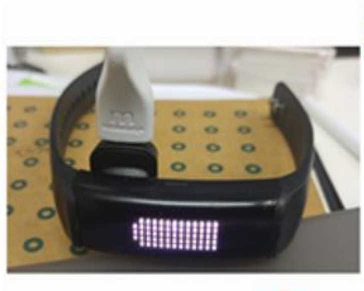

Smartband
CROISE.S
Activity tracker

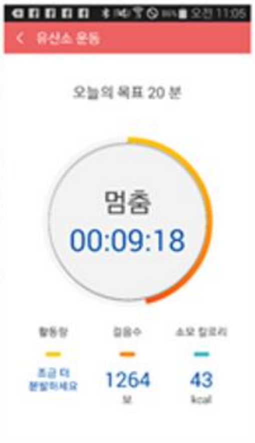

Sphygmomanometer and
blood pressure screenshot

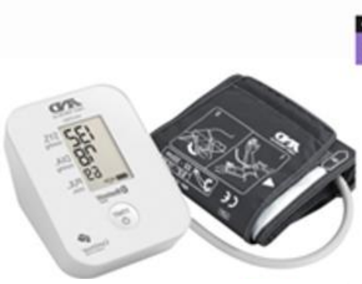

Sphygmomanometer
A&D

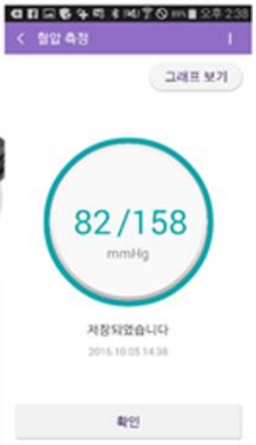

Glucose meter and
blood glucose saving screenshot

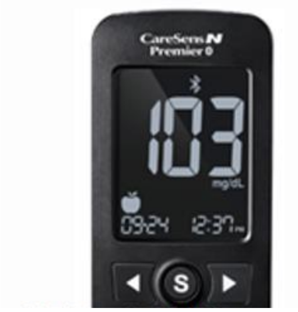

Glucose meter
CareSens N

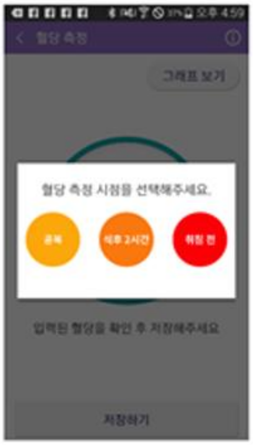

Supplement: Multimedia Appendix 1 [file jmir_v22i2e15377_app1.pdf]
