# Supplementary material for: Remote Management of Poststroke Patients With a Smartphone-Based Management System Integrated in Clinical Care: Prospective, Nonrandomized, Interventional Study
Source: J Med Internet Res. 2020 Feb 27;22(2):e15377. doi: 10.2196/15377 (PMC7068458; doi:10.2196/15377)
Supplement: Multimedia Appendix 2 [file jmir_v22i2e15377_app2.pdf]

Multimedia appendix 2. Clinical assessment of outcomes in this study

Clinical outcomes	Purpose	Description
Stroke awareness score (total, %)	Awareness of stroke	Percentage scores of questionnaire (4 parts: definition of stroke, risk factors of stroke, treatment of stroke, and action plan against stroke)
Beck's depression inventory (BDI)	Depression scale	Self-questionnaire
EuroQol-5 Dimensions (EQ-5D)	Health-related quality of life	Self-questionnaire
Physical measurements	Objectives related with stroke risk	Systolic blood pressure, diastolic blood pressure, body mass index, waist circumference
