# Supplementary material for: Remote Management of Poststroke Patients With a Smartphone-Based Management System Integrated in Clinical Care: Prospective, Nonrandomized, Interventional Study
Source: J Med Internet Res. 2020 Feb 27;22(2):e15377. doi: 10.2196/15377 (PMC7068458; doi:10.2196/15377)
Supplement: Multimedia Appendix 3 [file jmir_v22i2e15377_app3.pdf]

Multimedia appendix 3. Questionnaire of the stroke awareness score in English

Part 1. Definition of stroke (11 points)	Answer
1. Have you ever heard of “stroke” before? (No point)	1
1) Yes 2) No	
2. Which kind of disease do you think is “stroke”? (1 point)	2
1) Aging and degenerative disease of brain	
2) Vascular disease of brain	
3) Convulsive disease of brain	
4) Heart disease	
5) Neural or muscle disease of arms and legs	
6) I don’t know	
3. Please choose the symptom of stroke. (Each question has an answer.) (10 points, 1 point for each question)	
(1) 1) Paralysis of one side of arm and leg 2) stiffness in neck	1
(2) 1) Chest pain 2) language problem (words do not come out or do not understand other’s words)	2
(3) 1) hand tremble 2) loss of sense in one side of body	2
(4) 1) diplopia (an object seen like two) 2) tinnitus (ears ringing)	1
(5) 1) visual problem (one side of scene is not seen) 2) eyerid tremble	1
(6) 1) back pain 2) articular problem (cannot pronounce clearly)	2
(7) 1) Facial paralysis 2) All hands and feet numbness	1
(8) 1) consciousness problem 2) breathing problem	1
(9) 1) dry eye 2) gait difficulty	2
(10) 1) visual loss in one eye 2) cold sweat	1

Part 2. Risk factors of stroke (10 points)	
4. Please choose 10 risk factors of stroke. (10 points, 1 point each)<br>1) smoking 2) binge drinking 3) hypertension<br>4) stress 5) high cholesterol 6) low economic status<br>7) obesity 8) physical inactive 9) family history of stroke<br>10) diabetes 11) old age 12) contraceptive medicine<br>13) heart disease including arrhythmia 14) liver disease 15) kidney disease	1, 2, 3, 5,<br>7, 8, 9,<br>10, 11, 13
5. Have you ever heard of “thrombolysis treatment” which is the way of treatment for acute phase of ischemic stroke (brain infarction)? (5 points)<br>1) Yes (go to 5-1)<br>2) No (go to 6)	1
Part 3. Treatment of stroke (8 points)	
5-1. When you can have “thrombolysis treatment”? (1 point)<br>1) If you only go to a big hospital<br>2) If you go to hospital right away when you feel symptoms<br>3) If oriental medicine treatment is not effective<br>4) Whenever if you are diagnosed as a stroke<br>5) I don’t know	2
6. Does stroke recur well? (1 point)<br>1) Yes 2) No 3) I don’t know	1
7. When do you have to take medicine if you are diagnosed as stroke? (1 point)	4

1) During admission

2) During symptoms relieving

3) During 1 year regardless of symptoms

4) During life regardless of symptoms

Part 4. Action plan against stroke

8. What will you do when symptoms of stroke occur? 4

   1) Visit a general hospital

   2) Wait until the symptom got better

   3) Visit an oriental medicine clinic or oriental medicine hospital

   4) Call 911 and am ambulance

   5) Visit a hospital where you visited before

9. Which do you think beneficial folk remedies for stroke before arriving at a hospital? 6

   1) Stab hands or feet with a needle

   2) Take an oriental medicine (Cheongshimhwan)

   3) Take medicines previously described

   4) Massage paralyzed arm and leg

   5) Eat a meal

   6) No helpful remedies
