# Supplementary material for: Remote Management of Poststroke Patients With a Smartphone-Based Management System Integrated in Clinical Care: Prospective, Nonrandomized, Interventional Study
Source: J Med Internet Res. 2020 Feb 27;22(2):e15377. doi: 10.2196/15377 (PMC7068458; doi:10.2196/15377)
Supplement: Multimedia Appendix 4 [file jmir_v22i2e15377_app4.pdf]

Multimedia appendix 4. Demographic characteristics and clinical information of study participants

	All participants (n=99)
Sex	
Male	60 (61%)
Female	39 (39%)
Age	57.9 ± 10.8
Stroke subtype	
Ischemic	61 (62%)
Hemorrhagic	38 (38%)
Intracerebral	30 (30%)
Subarachnoid	8 (8%)
Clinical information	
Height	163.6 ± 8.8
Weight	66.0 ± 10.9
Body mass index	24.7 ± 3.1
Waist circumference	66.0 ± 10.9
Hypertension	71 (72%)
Diabetes mellitus	20 (20%)
Dyslipidemia	32 (32%)
Vital signs	
Systolic blood pressure	125.0 ± 17.5
Diastolic blood pressure	82.3 ± 9.8
Heart rate	74.0 ± 11.8

[a] Continuous variables were described as mean ± 1standard deviation (1SD).
