# Supplementary material for: Remote Management of Poststroke Patients With a Smartphone-Based Management System Integrated in Clinical Care: Prospective, Nonrandomized, Interventional Study
Source: J Med Internet Res. 2020 Feb 27;22(2):e15377. doi: 10.2196/15377 (PMC7068458; doi:10.2196/15377)
Supplement: Multimedia Appendix 5 [file jmir_v22i2e15377_app5.pdf]

Multimedia appendix 5. Changes in the total stroke awareness score and the scores of each part using smartphone-based management system

Main outcomes	Visit 1 (0 week)	Visit 2 (4 weeks)	Visit 3 (12 weeks)	Visit 2-Visit 1		Visit 3- Visit 1		SE
				Value	P-value	Value	P-value	
Stroke awareness score (total, %)	59.6 ± 18.1	67.6 ± 16.0	74.7 ± 14.0	7.98	<.001	15.12	<.001	1.30
Scores of each part								
Definition and symptoms of stroke (%)	74.3 ± 17.8	76.8 ± 18.7	78.8 ± 16.3	2.48	.13	4.50	.0066	1.64
Risk factors of stroke (%)	52.5 ± 26.8	64.2 ± 23.1	74.6 ± 24.0	11.72	<.001	22.12	<.001	2.62
Treatment of stroke (%)	52.4 ± 38.7	63.3 ± 34.8	75.1 ± 29.9	10.86	.0010	22.73	<.001	3.25
Immediate actions against stroke (%)	42.9 ± 37.1	51.0 ± 36.3	51.0 ± 35.7	8.08	.034	8.08	.034	3.79

[a] Scores were described as mean ± 1standard deviation (1SD). Abbreviation: SE (Standard Error)

[b] Scores of visit 1 was compared with scores of visit 2 and visit 3 with repeated measure analysis of variance (RM-ANOVA)
