# Supplementary material for: Remote Management of Poststroke Patients With a Smartphone-Based Management System Integrated in Clinical Care: Prospective, Nonrandomized, Interventional Study
Source: J Med Internet Res. 2020 Feb 27;22(2):e15377. doi: 10.2196/15377 (PMC7068458; doi:10.2196/15377)
Supplement: Multimedia Appendix 6 [file jmir_v22i2e15377_app6.pdf]

Multimedia appendix 6. Changes in Beck's depression inventory (BDI) scores among patients who were depressed and not depressed, and in the EuroQol-5 Dimensions (EQ-5D) scores

Main outcomes	Visit 1 (0 week)	Visit 2 (4 weeks)	Visit 3 (12 weeks)	Visit 2-Visit 1		Visit 3- Visit 1		SE
				Value	P-value	Value	P-value	
BDI	12.7 ± 10.1	11.2 ± 10.2	10.7 ± 10.2	-1.57	.011	-2.07	<.001	0.61
BDI, depressed (n=40)	23.2 ± 6.6	19.8 ± 8.8	19.6 ± 9.1	-3.35	.0028	-3.63	.0013	1.08
BDI, not depressed (n=59)	5.6 ± 4.2	5.3 ± 6.0	4.6 ± 5.1	-0.36	.61	-1.02	.15	0.69
EQ-5D	0.66 ± 0.33	0.69 ± 0.34	0.69 ± 0.34	0.025	.21	0.029	.14	0.020

[a] Percent scores were described as mean ± 1standard deviation (1SD). Abbreviation: SE (Standard Error).

[b] Scores of visit 1 was compared with scores of visit 2 and visit 3 with repeated measure analysis of variance (RM-ANOVA).
