# Supplementary material for: Remote Management of Poststroke Patients With a Smartphone-Based Management System Integrated in Clinical Care: Prospective, Nonrandomized, Interventional Study
Source: J Med Internet Res. 2020 Feb 27;22(2):e15377. doi: 10.2196/15377 (PMC7068458; doi:10.2196/15377)
Supplement: Multimedia Appendix 7 [file jmir_v22i2e15377_app7.pdf]

Multimedia appendix 7. Changes in blood pressure, body mass index and waist circumference

Main outcomes	Visit 1<br>(0 week)	Visit 2<br>(4 weeks)	Visit 3<br>(12 weeks)
Uncontrolled BP (n=26)			
SBP, mmHg	145.5 ± 15.6	134.2 ± 16.0	131.5 ± 12.4
DBP, mmHg	91.3 ± 8.6	84.6 ± 9.7	85.2 ± 8.5
Normal BP (n=73)			
SBP, mmHg	117.7 ± 11.4	122.2 ± 12.8	123.3 ± 14.3
DBP, mmHg	79.0 ± 8.0	79.9 ± 8.7	80.3 ± 9.7
Total BP (n=99)			
SBP, mmHg	125.0 ± 17.5	125.4 ± 14.6	125.5 ± 14.3
DBP, mmHg	82.3 ± 9.8	81.1 ± 9.2	81.6 ± 9.6
BMI	24.7 ± 3.1	24.7 ± 3.1	24.7 ± 3.1
Waist circumference	91.6 ± 7.6	92.0 ± 7.8	92.2 ± 7.6

Main outcomes	Visit 2-Visit 1		Visit 3- Visit 1		SE
	Value	P-value	Value	P-value	
Uncontrolled BP (n=26)					
SBP, mmHg	-11.27	.0010	-13.92	<.001	3.21
DBP, mmHg	-6.73	<.001	-6.19	<.001	1.60
Normal BP (n=73)					
SBP, mmHg	4.49	.013	5.58	.0021	1.78
DBP, mmHg	0.84	.45	1.29	.25	1.10
Total BP (n=99)					
SBP, mmHg	0.35	.83	0.45	.79	1.68

DBP, mmHg	-1.15	.23	-0.68	.95	0.48
BMI	0.060	.43	0.014	.85	0.076
Waist circumference	0.13	.37	0.15	.32	0.15

[a] Percent scores were described as mean ± 1standard deviation (1SD). Abbreviation: BP (blood pressure), SE (Standard Error).

[b] Scores of visit 1 was compared with scores of visit 2 and visit 3 with repeated measure analysis of variance (RM-ANOVA).
